# A Raman spectral reference library of potential anthropogenic and biological ocean polymers

**DOI:** 10.1038/s41597-022-01883-5

**Published:** 2022-12-24

**Authors:** Emily A. Miller, Kevan M. Yamahara, Chris French, Neil Spingarn, James M. Birch, Kyle S. Van Houtan

**Affiliations:** 1 Monterey Bay Aquarium Research Institute, 7700 Sandholdt Rd, Moss Landing, California 95039 USA; 2 Monterey Bay Aquarium, 886 Cannery Row, Monterey, California 93940 USA; 3 S & N Labs, 2021 E. Fourth Street, Suite 112, Santa Ana, California 92705 USA; 4 Nicholas School of the Environment, Duke University, Durham, North Carolina 27708 USA

**Keywords:** Environmental impact, Research data

## Abstract

Microplastics have been extensively documented in marine ecosystems and food webs with devastating impacts. To solve this global crisis, identifying the polymer composition is key for resolving the material origin, geographic source, and ecosystem life cycle of ocean plastics. Visually based techniques, importantly, are not diagnostic. Raman spectroscopy is an increasingly preferred identification method for its accuracy and reduced likelihood of misinterpretation, though it can be inaccessible due to cost of paywalled spectral libraries and availability of relevant polymer spectra for comparison. Here, we provide an open-access reference library of high-quality, broad-spectrum Raman spectra of major polymer categories germane to marine environments. The library includes high-quality spectra from: (a) pristine anthropogenic polymers newly sourced from manufacturers (*n* = 40), (b) weathered anthropogenic polymers collected from used consumer, beachcast, agricultural, and fishery sources (*n* = 22), and (c) biological polymers representing diverse marine taxa, trophic levels, and tissues (*n* = 17). We hope this reference library can help this rapidly expanding scientific community and facilitate progress in the global plastic pollution crisis.

## Background & Summary

With the discovery of marine microplastic pollution, studies examining the source and fate of this debris have increased by 10-fold in the last decade^1^. Microplastics (<5 mm and >0.1 μm)^2^ have been documented across remote and diverse marine ecosystems^3,4^, in taxa from all trophic levels^5–8^, across all organismal life stages^9–11^, as well as within human tissues, including the placenta^12^. A primary objective of these studies is to describe microplastic pervasiveness and to highlight the pressing environmental and health concern posed. After recording microplastic presence, a frequent objective is to determine the source and subsequent pathway of the debris to mitigate this ocean pollution^13–15^. Researchers use a suite of techniques to identify the polymer material to ascertain its source. However, frequently used affordable techniques such as visual identification, staining, and melting tests are not diagnostic of polymer specificity^13^. Additionally, increasingly smaller microplastics, including nanoplastics (<0.1 μm), are becoming the focus of research adding to the challenge of these identification approaches.

Vibrational spectroscopy approaches like Raman spectroscopy are increasingly popular methods of polymer identification^16^. Raman spectroscopy measures the shift in frequency of scattered light when a laser is directed at a sample. The shift is specific to the interaction between the laser and the vibrational energy of the molecules in the sample. A related vibrational approach, Fourier-transform infrared (FTIR) spectroscopy, directs a broad-spectrum infrared source at the sample but instead records the light remaining from the original light source after reflecting off of or passing through the sample. Using these methods, spectra from an unknown material can be matched to spectra in a library of known materials to identify molecular structure^17^. Raman and FTIR can be used on complementary molecular structures and sizes, and these spectroscopy approaches more accurately identify microplastic presence than visual assessment^13^, especially at smaller particle sizes. Spectroscopy methods also avoid identification biases related to particle morphology and tensile character that impact visual methods^18^. By contrast to transmission FTIR, Raman spectroscopy methods are non-destructive, and do not affect sample specimens.

Though increasingly popular, Raman spectroscopy is not widespread in microplastic research in part due to the technical expertise required (e.g., to mitigate spectral distortion from fluorescence) and the expense of operating specialized equipment. Because of these requirements, samples are often outsourced to third-party diagnostic laboratories. These laboratories typically have spectral libraries of common pristine polymers for spectral matching. Alternatively, if researchers have access to spectroscopy equipment, Raman spectral databases exist online for spectral matching but are locked behind expensive paywalls with annual subscriptions^19,20^. The costs of processing along with spectral matching make both these approaches out of reach for many academic researchers. Furthermore, many such laboratories and databases lack potential polymers specific to the marine environment in addition to anthropogenic polymers that have undergone environmental weathering. Such plastic polymer libraries are only beginning to become publicly available^21^, and open access biological spectra are often limited to specific taxa that are not relevant to marine systems^22–24^.

Our effort here, and that of others, to advance the identification of polymer species can draw on the practical lessons from the DNA barcoding revolution of the early 2000s. To maximize the promise and minimize the pitfalls of applying Raman spectroscopy to the global plastic pollution crisis, we recommend establishing open access libraries, clearly defining the applied use of spectral data, developing strict quality control standards, and encouraging contributions from a broad user community^25–28^.

Here, we present a database^29^ of Raman spectra derived from a broad suite of polymers potentially present in the marine environment. We include pristine and weathered anthropogenic polymers, as well as biological polymers from diverse taxa, trophic levels, and tissue structures. By including biological polymers, we provide non-target data (non-plastic) in addition to target data (plastic), often an overlooked approach which facilitates greater accuracy of broad category assignments. For example, a recent study documented that while 63% of Pacific oysters (*Crassostrea gigas*) contained microparticles, only an estimated 2% of those particles were anthropogenic plastics. The rest of the identified particles were comprised of: cellulose, calcium carbonate, protein, or other such natural biological component^30^. If non-target biological spectra are not included in reference libraries, a nearest match with an anthropogenic polymer will present false positives and overestimate the extent of plastic pollution. In our analytical code, we present a matching protocol that returns the degree of spectral matching of unlabeled spectra with labeled spectra, which can be used for quantitative comparisons. Our objective is to provide an open-access reference library and statistical routine for spectral post-processing and matching for the marine pollution research community to use in identifying microplastics in aquatic ecosystems.

## Methods

**Fig. 1:**
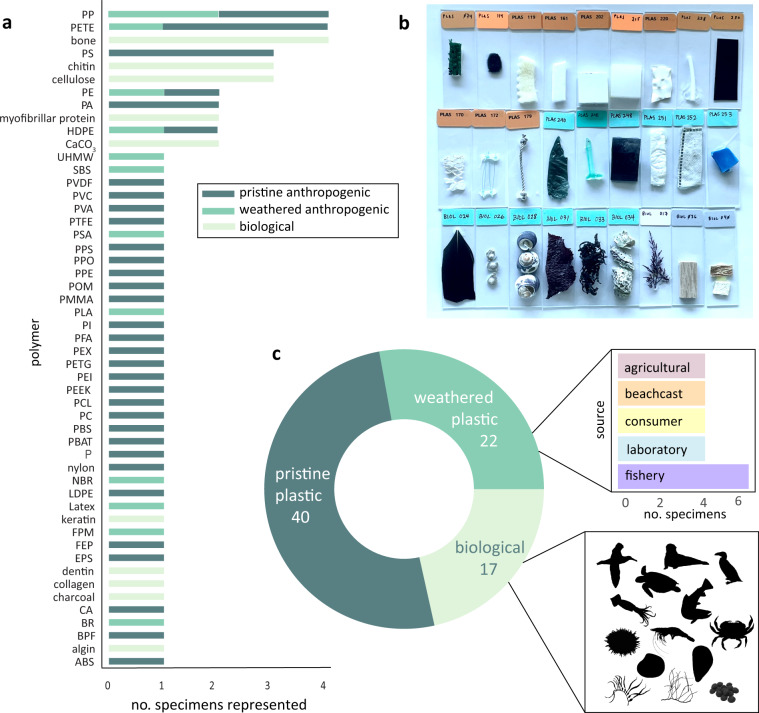
The reference library contains spectra encompassing common polymers present in the marine environment. (**a**) Counts of specimens representing each polymer type. Colors reflect the type of specimen represented for each polymer: pristine anthropogenic, weathered anthropogenic, and biological polymers. (**b**) Photo of mounted specimens in the library including pristine anthropogenic polymers (top row), weathered anthropogenic polymers (middle), and biological polymers (bottom). (**c**) The library consists of polymers broken down by category. Bar plot (top inset) breaks down the sources of weathered polymers. Silhouettes (bottom inset) represent the taxa present in the biological polymers.

We populated the reference library^29^ with representative anthropogenic and biological polymers from major polymer categories^4,31,32^. We described the diversity of these specimens using several parameters (Table 1). We obtained pristine anthropogenic samples from plastics manufacturers, newly purchased consumer products, and fishing gear from commercial vendors in Monterey County, CA (*n* = 40, Table S1). Weathered anthropogenic polymers consisted of used consumer (*n* = 4, Monterey County, CA), fishery (*n* = 6, fishermen working in Monterey Bay, CA), agricultural (*n* = 4, collected along the Salinas River and tributaries, Monterey County, CA), laboratory (*n* = 4, used in the processing laboratory, the Ocean Memory Laboratory, Monterey Bay Aquarium, CA), and beachcast specimens (*n* = 4, southern Monterey Bay, CA; Fig. 1). Biological polymers were obtained from the Monterey Bay Aquarium’s archived collections which originated from Monterey Bay, CA (*n* = 17, Table S1; Fig. 1).

**Table 1:** Explanation of terms used in reference library metadata.

term	definition
unique_id	Identifier of each specimen.
color	Color description of specimen.
poly_acronym	International polymer acronym.
polymer	Polymer name.
structure	Physical structure or form of specimen.
category	One of three polymer categories: pristine anthropogenic, weathered anthropogenic, or biological.
description	A description of the specimen and its source.
parent_grp	Parent group description: biological, industrial, consumer, laboratory, beachcast, agricultural, and fishery.
location_collected	For biological and weathered anthropogenic specimens, the general location the specimen was collected if known.

Polymer specimens were sectioned or cut depending on size and shape to fit on standard optical glass slide (25.4 × 76.2 × 1 mm, No. BS-72P, AmScope). Specimens were affixed to the slides using a hot glue gun (No. W099029AE, WORKPRO) and resin hot glue (ethylene-vinyl acetate, No. W133233A, WORKPRO). If we collected multiple specimens from a polymer category, those selected for mounting differed by color or opacity. We followed a strict quality control protocol to avoid any cross contamination polymers.

**Fig. 2:**
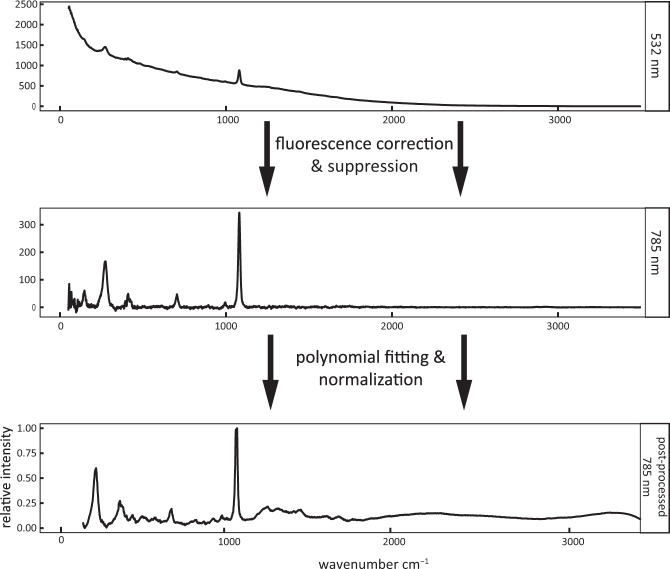
Raman spectra were corrected and post-processed for use in the library. All spectra were corrected for fluorescence and scanned at 532 nm on a DXR Raman Microscope (Thermo Scientific, USA; top panel). For those specimens still requiring it, fluorescence was suppressed by scanning at 785 nm on an XploRA PLUS Confocal Raman Microscope (HORIBA Scientific, USA) (middle panel). This step was not required for most specimens. Each specimen was scanned 100 times and LabSpec 6 Spectroscopy Suite software (Horiba Scientific, USA) generated a single averaged spectrum. Spectra were then fit with a polynomial model, SNV normalized, and rescaled on a 0–1 relative intensity scale (bottom panel). These steps corrected and suppressed fluorescence, removed background noise and filled missing data, and allowed spectra to be compared across specimens.

S&N Labs (CA, USA) analyzed mounted polymer specimens using Raman spectroscopy (DXR Raman Microscope, Thermo Scientific, USA). Samples were first run at 532 nm wavelength (8.7 mW, 5.5–8.3 cm^−1^ resolution, 50x objective) and corrected for background fluorescence as needed (Fig. 2). If a spectrum could not be acquired due to high fluorescence, then it was analyzed using comparable power selection and resolution parameters at 785 nm wavelength (XploRA PLUS Confocal Raman Microscope, HORIBA Scientific, USA; Fig. 2). They were each scanned 100 times and averaged.

The averaged spectrum output was processed using a statistical routine script (R Statistical Software v3.6.3)^33,34^ that included a median filter window, polynomial fitting, normalization, and rescaling (Fig. 2); following established protocols^4^. Spectra were processed using a 15 wavenumber-wide median filter window to remove background noise. Denoised spectra were fit to a seventh order polynomial model served to perform baseline correction to accommodate sample variation^35^. The polynomial fitting provides continuous spectral intensities which allows for comparison at each wavenumber without missing data. These steps required R packages fda, hyperSpec, pspline, and signal^36–39^. Standard Normal Variate (SNV) normalization then allowed for spectra across samples to be compared. To compare sample spectra, we transformed spectra intensity values using SNV normalization, and rescaled values from 0–1 to be compare across wavelengths.

**Fig. 3:**
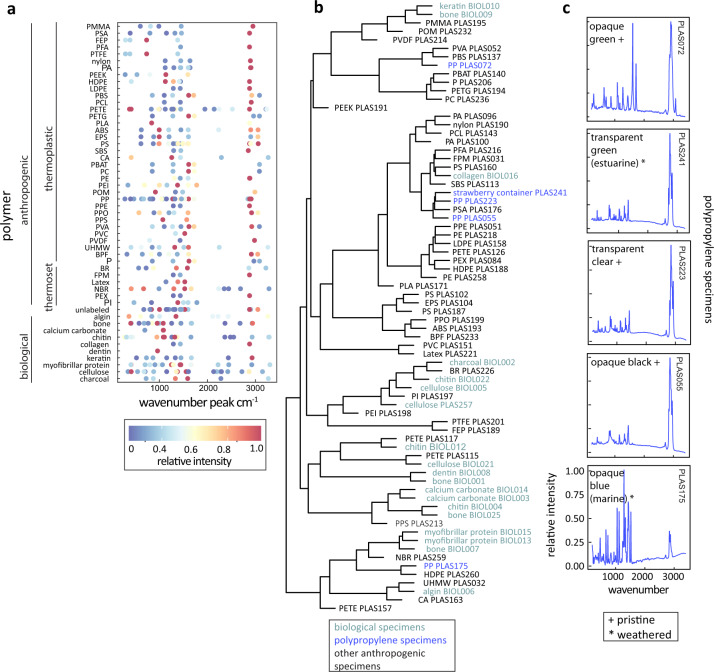
Reference library spectra convey polymer relationships and patterns. (**a**) The wavenumber peaks for each polymer type are shown with color indicating relative intensity. Polymers are categorized by biological and anthropogenic sources and grouped by polymer type within those categories. (**b**) A cluster dendrogram showing similarities between specimen spectra. The hierarchical cluster analysis uses a Pearson’s distance matrix calculated between each pair of specimen spectra. Biological specimens are written in green and polypropylene specimens in blue. (**c**) Spectra for all polypropylene specimens within the library are visualized, demonstrating the effects of weathering, color, opacity, and individual sample on spectra variability. These spectra panels include three pristine and two weathered specimens, noted by (+) and (*), respectively. One weathered specimen (PLAS241) is unlabeled and sourced from a strawberry basket found in the Salinas River estuary, connecting to the Monterey Bay. Based on common industry manufacturing practices, this basket sample is likely polypropylene.

**Fig. 4:**
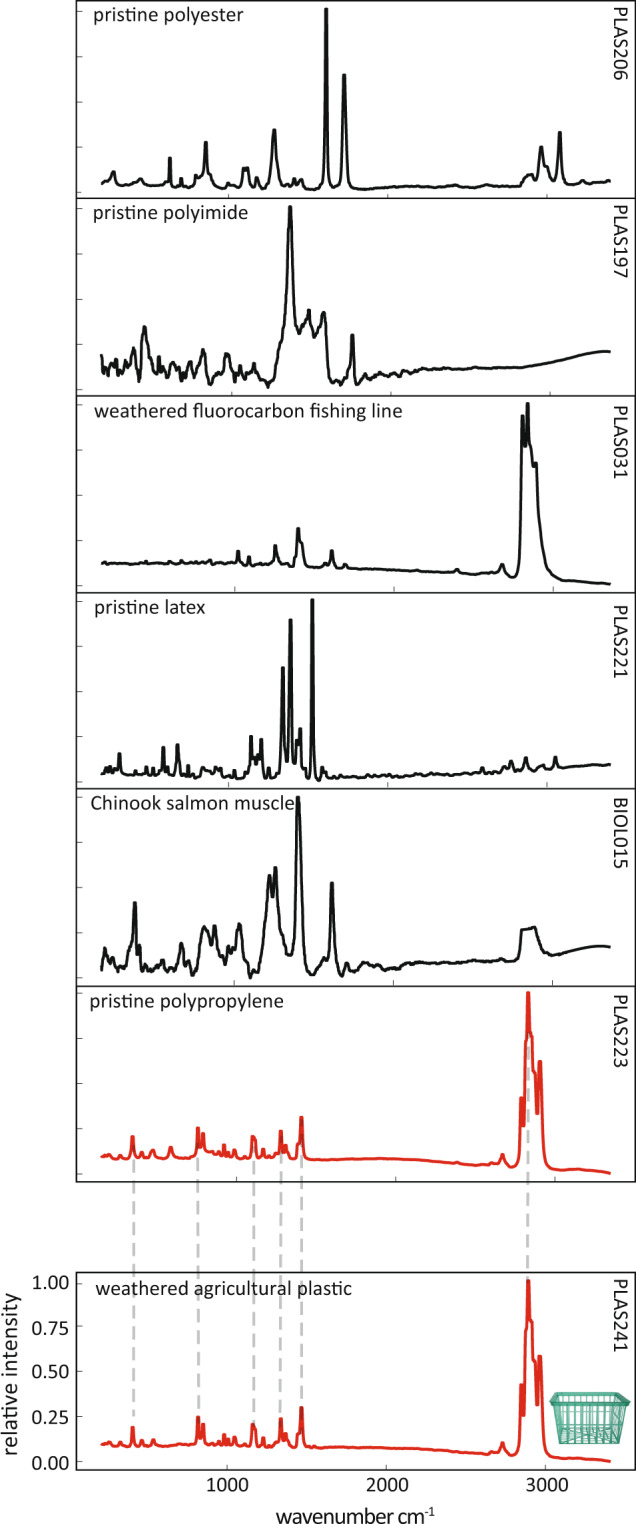
Spectra of unknown plastics found in the environment can be matched to known spectra in the reference library. The spectrum from a weathered strawberry basket (red, bottom) collected from an agricultural region of the Salinas River watershed matched with spectrum of a pristine polypropylene specimen (red, second to bottom), and was confirmed using S&N’s plastics library. In contrast, the strawberry basket spectrum did not covary with other spectra in the library. Example spectra from pristine and weathered anthropogenic polymers and a biological polymer are displayed in top panels for comparison. Gray dashed lines denote matched spectral peaks for illustrative purposes - matching was done quantitatively using the Pearson distance matching protocol (see Methods).

Once post-processed, we converted all spectra into vectors along the 200–3400 cm^−1^ wavenumber range. Each vector, whether labeled or unlabeled, can be matched against all other vectors from the labeled reference library. We compared labeled, library specimen spectra against one another to determine polymer family relatedness (Fig. 3) and used this protocol to match unlabeled, environmental spectra to known reference library spectra (Fig. 4). After turning each spectrum into a vector, our protocol matched a focal vector against each spectrum in the library and calculated the Pearson’s correlation coefficient (*r*) for each pair. The protocol generates a matrix of all Pearson’s correlation coefficients between pairs and selects the minimum value to identify matching pairs for assignment if unlabeled, or for dendrogram construction if comparing all labeled specimens.

## Data Records

Raw data records of the reference library are located in the online repository (https://osf.io/7cqv4/)^29^ in one folder of raw spectra (“Data1_raw_csv.zip”) and one separate file of post-processed spectra (“Data2_processed.csv). The raw data include Raman spectra of 79 specimens in non-proprietary .csv file format (“Data1_raw_csv.zip”). These broad-spectrum data records include target specimens: pristine anthropogenic polymers (*n* = 40) and weathered anthropogenic polymers (*n* = 22); and also include non-target specimens of biological polymers (*n* = 17). If more than one usable spectrum was obtained for a given sample, they were included files (*n* = 87 spectra). Processed spectra, processed using the above methods, are also included in .csv format (“Data2_processed.csv”). Metadata terms are defined in Table 1. Metadata itself is in Table S1. Visualized processed spectra from Data2_processed.csv are located in Supplemental Information, Figure S1. Despite concerted efforts, we could not obtain spectra from 15 specimens (Table 2), and these are excluded in our library sample counts.

**Table 2:** Metadata of polymer specimens for which spectra could not be obtained.

entry no.	sample_id	color	poly_acronym	polymer	structure	category	description	parent_grp	location_collected
1	BIOL011	brown	—	keratin	fragment	biological	hawksbill turtle, *Eretmochelys imbricata*, scute	biological	Hawaii
2	BIOL018	red	—	algin	sheet	biological	*Cryptopleura* sp. algae blade	biological	Monterey Bay, CA
3	BIOL019	green	—	algin	sheet	biological	*Ulva* sp. algae blade	biological	Monterey Bay, CA
4	BIOL020	brown	—	cellulose	sheet	biological	cardboard box	biological	commercial
5	BIOL026	white	—	calcium carbonate	fragment	biological	Pacific purple urchin, *Strongylocentrotus purpuratus*, shell	biological	Monterey Bay, CA
6	BIOL027	brown	—	keratin	fur	biological	sea otter, *Enhydra lutris*, pup pelt SO # 1971–91	biological	Monterey Bay, CA
7	BIOL029	gray, red before drying	—	algin, calcium carbonate	fragment	biological	*Corallina* sp. calcareous red algae	biological	Monterey Bay, CA
8	BIOL030	brown green	—	algin	sheet	biological	Egregia menziesii, feather boa kelp	biological	Monterey Bay, CA
9	BIOL032	dark green brown	—	algin	fragment	biological	*Nereocystis luetkeana*, bull kelp neumatocyst	biological	Monterey Bay, CA
10	PLAS039	black	PMMA	acrylic	sheet	pristine	optix acrylic 2025, alpha packaging	consumer	commercial
11	PLAS053	clear	PE	polyethylene	sheet	pristine	costume bag	consumer	commercial
12	PLAS076	black	PVC	vinyl	flooring	pristine	Vinyl flooring sample	building material	commercial
13	PLAS192	gray	CPVC	chlorinated polyvinyl chloride	sheet	pristine	McMaster Carr samples	industrial	commercial
14	PLAS196	gray	PAI	polyamide-imide	sheet	pristine	Torlon® samples	industrial	commercial
15	PLAS229	amber	MDI	methylene diphenyl diisocyanate	resin	pristine	gorilla glue	consumer	commercial

## Technical Validation

To control for background fluorescence, we performed fluorescence detrending using a baseline correction and cosmic ray correction (LabSpec 6 Spectroscopy Suite software, Horiba Scientific, USA). If this did not remedy the fluorescence observation, the alternate laser excitation level was used. Each specimen was scanned 100 times. These 100 scans were averaged to produce a single spectrum (LabSpec 6 Spectroscopy Suite software, Horiba Scientific, USA). Periodic system calibrations were performed using a polystyrene (Thermo Scientific microscope) or silicon standard (Horiba Scientific microscope) calibration slide. The laser was set to 0.1% power and focused with the 50x objective. When cool, the gratings were then auto-calibrated. Prior to running each new set, graphite was used as a signal level verification.

To visualize and explore the data here, we performed several basic analyses using post-processed spectra. We grouped spectra by polymer type and filtered for local maxima in spectra intensity (Fig. 3a). Wavenumbers ranged from 200–3400 cm^−1^ and local maximum were defined as the highest point within a moving window of 101 wavenumbers to minimize spectral noise. Additionally, we used a minimum intensity threshold of 0.2 (on a relative scale of 0–1).

We calculated the Pearson’s correlation coefficient between each pair of spectra, indicative of how closely each pair covaries^40^. With these correlation values we built a distance matrix of dissimilarity between all specimens in the library. Using this matrix we performed a hierarchical cluster analysis (stats package in R)^33^ to create a cluster dendrogram (Fig. 3b). The cluster dendrogram illustrates relatedness between polymers based on spectra similarity. Biological polymers grouped in several distinct groups, with anthropogenic polymers intermixed. We found relatively high variation within a given polymer family. An example of this variation is shown for polypropylene (Fig. 3c). Variation may be related to the gross structure (rough or smooth surface), the surface structure at a microscale (crystalline vs loose), color, opacity, reflectivity, or weathering.

We performed polymer assignment for eight unknown weathered anthropogenic specimens with our post-processing and matching routine using our reference library^29^ and with S&N’s Raman spectral library (*n* = ~6000 materials). We had identical assignments for 4 of the 8 unknowns. One weathered specimen, a transparent-green produce (strawberry) basket was collected from a Salinas River tributary, an agricultural watershed tidally influenced by the Monterey Bay, CA. Agricultural baskets such as this are commonly made from polypropylene. Using our matching routine and reference library^29^, the agricultural basket matched a pristine polypropylene specimen (Figs. 3b, 4). We confirmed this match as polypropylene using S&N’s Raman spectral library (Figure S2). Spectra of the agricultural basket, the matched pristine polypropylene, and example non-matching spectra from other common polymers illustrate the theory underpinning the peak matching process and emphasize the utility of this library in environmental microplastic research. For two of the differing assignments, S&N matched the spectra with pigment additives within the polymers. In the third, S&N assigned the material as polypropylene and an unknown organic additive and our reference library^29^ assigned the material as a pressure sensitive adhesive, many of which have a polypropylene component (Table S2).

These matching differences underscore the need for more open-access reference library specimens representing diverse colors within each polymer family as well as multiple polymer additive combinations common in manufacturing. Metadata must also include color descriptors, as we have here. Accompanying post-processing routine and matching protocols, such as presented here, are two additional aspects key to providing a reference library useful to the research community.

## Usage Notes

Researchers can use this reference library^29^, post-processing, and matching protocol to match unlabeled particles of unknown material composition to labeled spectra here (Fig. 3). This reference library^29^ will be useful in all environmental microplastic identification studies, though the selected polymers are established to be of significance to marine settings^4^. The polymer families represented in the library likely cover the majority of anthropogenic plastic debris found in the ocean^4^. It is especially suited to nearshore Pacific Ocean systems given the biological specimens chosen as well as the weathered anthropogenic polymers sourced from the coastal agricultural and fishery industries.

We have provided both the raw files produced by the Raman microscope and those we processed^29^ using the available script containing post-processing and matching routines^34^ to aid researchers in their microplastic labeling analyses. We have noted the spectra that could be obtained but were noisy (see asterisks in Table S1). These spectra should be used in combination with future repository contributions of the same polymer rather than on their own.

More spectra replicates are needed especially in certain focal areas. We observed spectral variation among specimens within a polymer type (Fig. 3b,c). Further contributions to online Raman repositories should include various spectra from each polymer type that represent known weathering time periods of increasing periods and suite of conditions. Marine weathering, for example, may degrade polymers at a different rate than freshwater weathering over the same duration. A controlled study with known weathering dates would address some of these weathering uncertainties^21^ and provide information about the temporal processes in microplastic debris pathways. In addition to weathering variability, polymer spectra of various colors, opacities, textures, species, and tissue types are needed. We observed differences in spectra between polymers of the same category and pristine status but differing color and potentially differing chemical additives. During our technical validation of unknown weathered specimens, we had several differing assignments due to pigments and additives. Anthropogenic polymer additives can affect spectra outputs and also should be considered in future applications^21,41^. Multiple spectra from each polymer that include these component variations will help researchers assign their unlabeled microparticles with greater precision. In addition to increasing specimen number and diversity, having an increased number of replicates of spectra from each specimen would allow for the generation of future machine learning routines that could produce more quantitative probabilistic matching to reference library spectra.

Raman spectral repositories are nearly all locked behind cost-prohibitive paywalls and subscriptions. The earlier advances of the DNA barcoding field provide practical lessons for these relatively nascent plastic polymer identification efforts. Like the expansive work of the DNA barcoding field, we hope that an era of spectral contributions to open access libraries from many users is beginning. This database^29^ compliments the few open-access repositories available^21^, contributing 24 new anthropogenic polymer types, and 18 non-target biological polymers previously unpublished in open-access spectral repositories (Table S3). Of the newly contributed weathered polymer types, two were sourced from marine fishing gear (FPM & UHMW), making them especially relevant to microplastic research in the marine environment. This broad-spectrum spectral reference library, post-processing routine, and matching protocol presented here will contribute to a growing open-access resource for microplastic researchers.

## Supplementary information

Supplementary Information

## Data Availability

Code for the spectral identification matching routine as well as the generation of figures for this paper are located on Github: https://github.com/emilymiller/spectra_microplas_reflib^34^. The script “user_instructions.txt” orients users to the spectra processing scripts titled, “cleaning_code.R” and “preprocessing.R”, followed by the spectral matching routine script, “technical_valid_weathered.R”.
