# Supplementary material for: A Raman spectral reference library of potential anthropogenic and biological ocean polymers
Source: Sci Data. 2022 Dec 24;9:780. doi: 10.1038/s41597-022-01883-5 (PMC9790010; doi:10.1038/s41597-022-01883-5)
Supplement: Supplementary file 1 — Supplementary Information [file 41597_2022_1883_MOESM1_ESM.pdf]

Supplementary Information

for

A Raman spectral reference library of potential anthropogenic and biological ocean polymers

AUTHORS: Emily Miller, Kevan Yamahara, Chris French, Neil Spingarn, Jim Birch, Kyle S. Van Houtan

Contents:

1. Figure S1. Unprocessed spectra of reference library specimens.
2. Figure S2. Example spectral matching using S&N's Raman spectral database to assign an unknown, weathered anthropogenic plastic to polypropylene.
3. Table S1. Metadata of polymer specimens in Raman reference library.
4. Table S2. Assignments for unknown weathered anthropogenic polymers using this reference library and a commercial Raman database (S&N Labs).
5. Table S3. Polymers new to open-access spectral repositories.

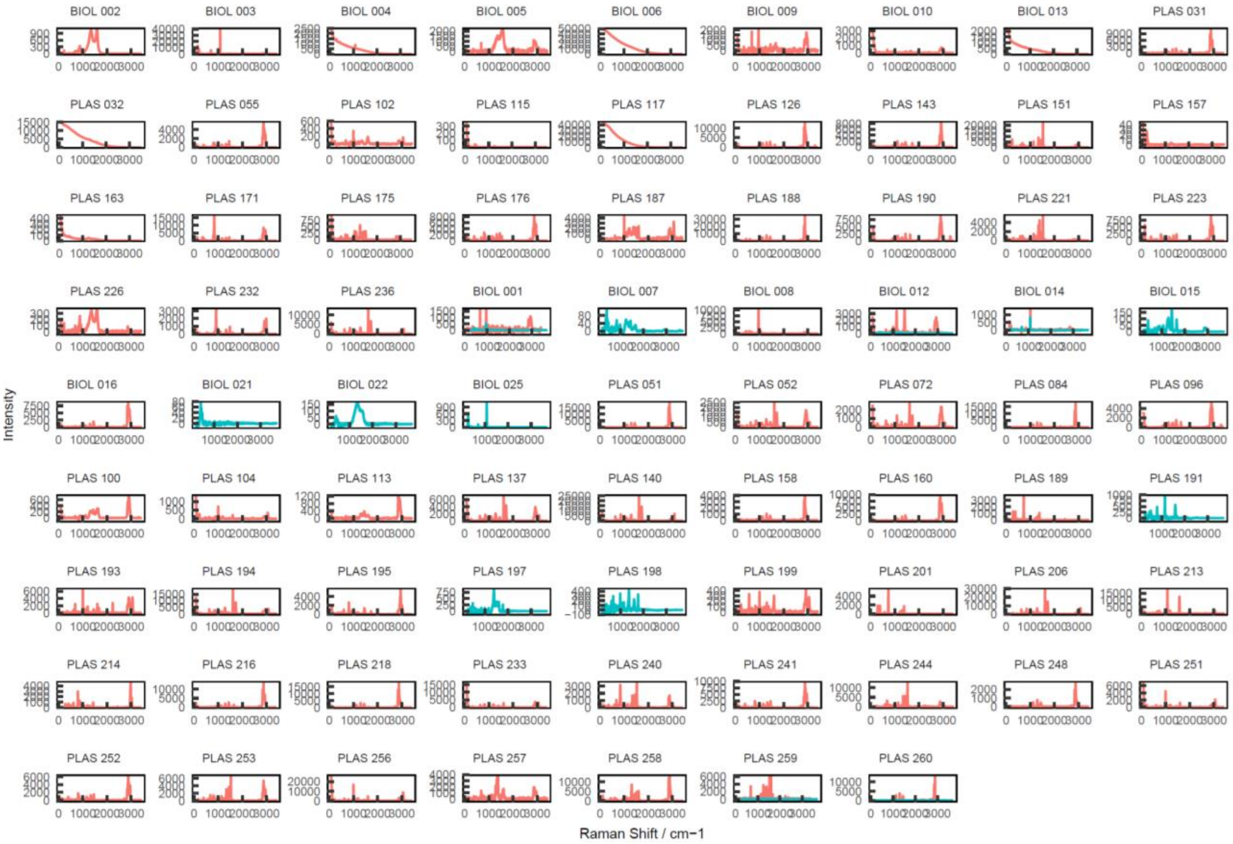

Figure S1. Unprocessed spectra of reference library specimens. Specimens were scanned at 532 nm, and if usable spectra could not be generated, at 785 nm. Spectra generated at 532 nm are shown in red and at 785 nm in blue. Y-axes various across plots.

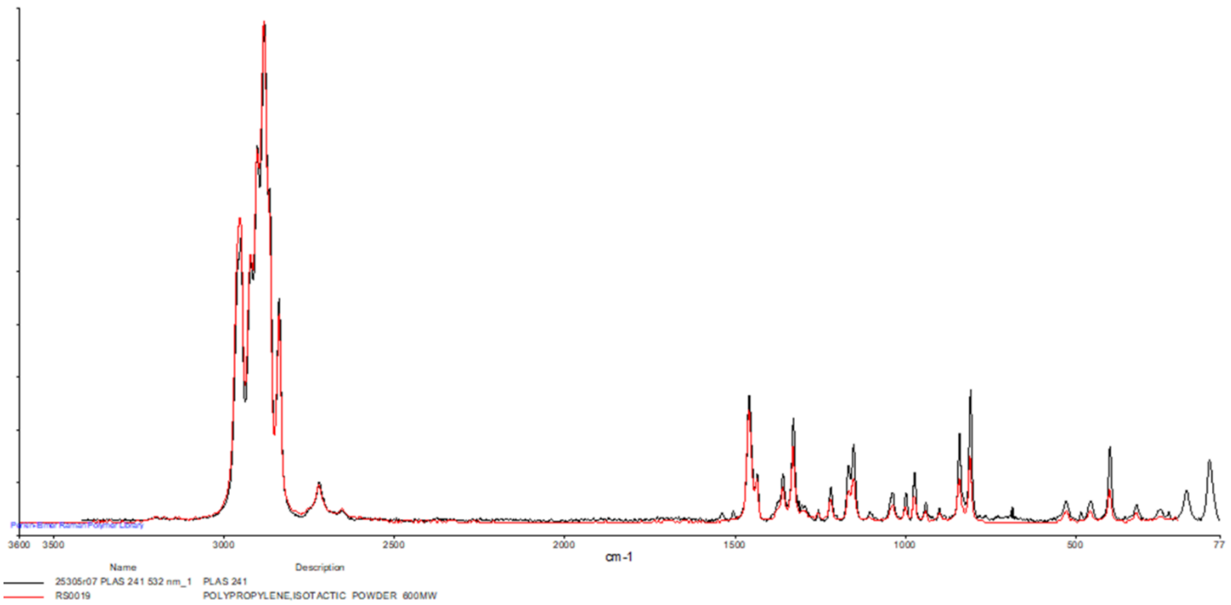

Figure S2. Example spectral matching using S&N's Raman spectral database to assign an unknown, weathered anthropogenic plastic to polypropylene. This assignment confirmed the assignment made using our matching routine and spectral reference library.

Table S1. Metadata of polymer specimens in Raman reference library. Spectra that could be obtained but were noisy are noted with an (*).

entry no.	unique_id	color	poly_acronym	polymer	structure	category	description	parent_grp	location_collected
1	BIOL001	white	--	bone	bone	biological	olive ridley sea turtle scapula condyle	biological	commercial
2	BIOL002	black	--	cellulose	fragment	biological	charcoal	biological	Monterey, CA
3	BIOL003	white	--	calcium carbonate	fragment	biological	surf clam shell	biological	Monterey Bay, CA
4	BIOL004*	purple brown	--	chitin	fragment	biological	dungeness crab carapace	biological	Monterey Bay, CA
5	BIOL005	dark green	--	cellulose	sheet	biological	*Zostera* sp. eelgrass blade	biological	Monterey Bay, CA
6	BIOL006*	brown green	--	algin	sheet	biological	*Macrocystis kelp* blade	biological	Monterey Bay, CA
7	BIOL007	white	--	bone	fragment	biological	rhino auklet upper mandible horn	biological	Monterey Bay, CA
8	BIOL008	white	--	dentin	fragment	biological	sea otter tooth	biological	Monterey Bay, CA
9	BIOL009	white	--	bone	fragment	biological	sea otter maxilla	biological	Monterey Bay, CA
10	BIOL010	gray	--	keratin	fragment	biological	Laysan albatross feather	biological	Monterey Bay, CA
11	BIOL012	brown	--	chitin	fragment	biological	tiger shrimp carapace	biological	market, Monterey, CA
12	BIOL013*	pink beige	--	myofibrillar protein	sheet	biological	market squid bell	biological	market, Monterey Bay, CA
13	BIOL014	iridescent white	--	calcium carbonate	fragment	biological	green mussel shell	biological	market, Monterey Bay, CA
14	BIOL015	orange	--	myofibrillar protein	muscle plug	biological	Chinook salmon muscle	biological	market, Monterey Bay, CA
15	BIOL016	gray	--	collagen	sheet	biological	Chinook salmon skin	biological	market, Monterey Bay, CA
16	BIOL021	brown	--	cellulose	rope	weathered	Choy et al 2019 gear #8, infrequently used 1/4" braided rope	fishery	Monterey Bay, CA
17	BIOL022	dark brown	--	chiton	fragment	biological	squid beaks, albatross stomach	biological	Monterey Bay, CA
18	BIOL025	purple	--	calcium carbonate	fragment	biological	Pacific purple urchin spines; Strongylocentrotus purpuratus; from otter female 613	biological	Monterey Bay, CA
19	PLAS031	clear	FPM	fluorocarbon rubber	fiber	weathered	Fluorocarbon 50 lb test	fishery	Monterey Bay, CA
20	PLAS032*	tan	UHMW	ultra high molecular weight polyethylene	fiber	weathered	spectra	fishery	Monterey Bay, CA
21	PLAS051	semi-clear	PPE	polyphenylene ether	sheet	pristine	wrapper	consumer	commercial
22	PLAS052	semi-opaque green	PVA	polyvinyl alcohol	sheet	pristine	Mater Bi ® starch-poly, Pull-N-Pak® bag	consumer	commercial
23	PLAS055	black	PP	polypropylene	lid	pristine	Penn tennis ball can lid	consumer	commercial
24	PLAS072	green	PP	polypropylene blend	sheet	pristine	Home Depot turf rug sample	consumer	commercial
25	PLAS084	white	PEX	cross-linked polyethylene	pipe	pristine	Plumbing, building material	consumer	commercial
26	PLAS096	white	PA	nylon	rope	pristine	West Marine nylon rope	fishery	commercial
27	PLAS100	black	PA	nylon	rope	pristine	West Marine nylon rope	fishery	commercial
28	PLAS102	black	PS	polystyrene	container	pristine	solo coffee lid	consumer	commercial

29	PLAS104	white	EPS	expanded polystyrene	packaging	pristine	Nespresso styrofoam box	consumer	commercial
30	PLAS113	black	SBS	poly(styrene butadiene styrene)	block	weathered	Lego tire	consumer	commercial
31	PLAS115	white	PETE	polyethylene terephthalate	fabric	pristine	SeattleFabrics/Malden Mills, PolarTec 200	consumer	commercial
32	PLAS117*	blue	PETE	polyethylene terephthalate	fabric	pristine	SeattleFabrics/Malden Mills, PolarTec 200	consumer	commercial
33	PLAS126	silver	PETE	polyethylene terephthalate	sheet	weathered	mylar balloon	consumer	commercial
34	PLAS137	white	PBS	polybutadiene styrene	disc	pristine	disc	bioplastic	commercial
35	PLAS140	white	PBAT	polybutylene adipate terephthalate	disc	pristine	disc	bioplastic	commercial
36	PLAS143	white	PCL	polycaprolactone	disc	pristine	disc	bioplastic	commercial
37	PLAS151	blue	PVC	polyvinyl chloride	tape	pristine	electrical tape, Commercial Electric	building material	commercial
38	PLAS157	black	PETE	polyethylene terephthalate	sheet	pristine	produce container, Cool-Pak	container	commercial
39	PLAS158	white	LDPE	low density polyethylene	foam	pristine	Lenovo packaging foam	electronics	commercial
40	PLAS160	clear	PS	polystyrene	glue	pristine	Shaxon high temperature mini glue sticks	adhesive	commercial
41	PLAS163	ivory	CA	cellulose acetate	fiber	weathered	cigarette butt, cellulose acetate	beachcast	Monterey Bay, CA
42	PLAS171	yellow	PLA	polylactic acid	rope	weathered	Choy et al 2019 gear #5, yellow, woven, flat, 3/8", fish traps, seldom used	fishery	Monterey Bay, CA
43	PLAS175	blue	PP	polypropylene	rope	weathered	Choy et al 2019 gear #10, cobalt blue	fishery	Monterey Bay, CA
44	PLAS176	blue	PSA	pressure sensitive adhesive	rope	weathered	Choy et al 2019 gear #11, braided, "blue steel" all purpose dock and trap lines	fishery	Monterey Bay, CA
45	PLAS187	red	PS	polystyrene	cup	pristine	solo cup	consumer	commercial
46	PLAS188	white	HDPE	high density polyethylene	bottle	pristine	pill bottle, high density polyethylene	consumer	commercial
47	PLAS189	white	FEP	fluorinated ethylene propylene	sheet	pristine	McMaster Carr samples	industrial	commercial
48	PLAS190	white	PA	nylon	sheet	pristine	McMaster Carr samples, cast nylon	industrial	commercial
49	PLAS191	tan	PEEK	polyether ether ketone	sheet	pristine	McMaster Carr samples	industrial	commercial
50	PLAS193	ivory	ABS	acrylonitrile butadiene styrene	sheet	pristine	McMaster Carr samples	industrial	commercial
51	PLAS194	clear	PETG	glycol-modified polyethylene terephthalate	sheet	pristine	McMaster Carr samples	industrial	commercial
52	PLAS195	white	PMMA	acrylic	sheet	pristine	Optix acrylic sheet sample	industrial	commercial
53	PLAS197	amber	PI	polyimide	sheet	pristine	McMaster Carr samples	industrial	commercial
54	PLAS198	amber	PEI	polyetherimide	sheet	pristine	Ultem® samples	industrial	commercial
55	PLAS199	black	PPO	polyphenylene oxide	sheet	pristine	NORYL, McMaster Carr samples	industrial	commercial
56	PLAS201	white	PTFE	polytetrafluoroethylene	sheet	pristine	McMaster Carr samples	industrial	commercial
57	PLAS206	white	P	polyester	sheet	pristine	McMaster Carr samples	industrial	commercial
58	PLAS213	ivory	PPS	polyphenylene sulfide	sheet	pristine	McMaster Carr samples	industrial	commercial
59	PLAS214	white	PVDF	polyvinylidene difluoride	sheet	pristine	McMaster Carr samples	industrial	commercial
60	PLAS216	clear	PFA	perfluoroalkoxy alkane	sheet	pristine	McMaster Carr samples	industrial	commercial
61	PLAS218	clear	PE	polyethylene	sheet	pristine	McMaster Carr samples	industrial	commercial

62	PLAS221	blue	latex	latex	sheet	weathered	balloon, latex	consumer	commercial
63	PLAS223	white	PP	polypropylene	sheet	weathered	drinking straw, polypropylene	consumer	commercial
64	PLAS226	black	BR	polybutadiene rubber	rubber	weathered	bicycle innertube, polybutadiene rubber	consumer	commercial
65	PLAS232	black	POM	polyoxymethylen e	pipe	pristine	Phister plumbing pipe, polyoxymethylene	industrial	commercial
66	PLAS233	white	BPF	bisphenol-F dihydroxydipheny lmethane	foam	pristine	Engineered Syntatic Systems Microsphere Syntactic Foam	industrial	commercial
67	PLAS236	gray	PC	polycarbonate	fiber	pristine	HBF textiles sample, polycarbonate	upholstery	commercial
68	PLAS240	green	--	--	sheet	weathered	green plastic mulch	agricultural	Salinas Valley watershed
69	PLAS241	green	PP	polypropylene	fragment	weathered	strawberry basket	agricultural	Salinas Valley watershed
70	PLAS244	blue	--	--	fragment	weathered	strapping tape blue	agricultural	Salinas Valley watershed
71	PLAS248	black	--	--	sheet	weathered	drip irrigation tubing	agricultural	Salinas Valley watershed
72	PLAS251	white	--	--	sheet	weathered	beachcast white foam sheet	beachcast	Pacific Grove, Monterey Bay
73	PLAS252	white, gray	--	--	sheet	weathered	white and gray mask filter	beachcast	Pacific Grove, Monterey Bay
74	PLAS253	blue	--	--	fragment	weathered	blue fragment	beachcast	Pacific Grove, Monterey Bay
75	PLAS256	clear	--	--	sphere	weathered	clear plastic hollow sphere	beachcast	Pacific Grove, Monterey Bay
76	PLAS257	blue	--	cellulose	woven fibers	weathered	blue cotton towel	oml laboratory	commercial
77	PLAS258	yellow	PE	polyethylene	sheet	weathered	yellow Tyvek suit	oml laboratory	commercial
78	PLAS259	green	NBR	acrylonitrile butadiene rubber	sheet	weathered	green nitrile glove	oml laboratory	commercial
79	PLAS260	white	HDPE	high density polyethylene	sheet	weathered	white underside Tyvek suit	oml laboratory	commercial

Table S2. Assignments for unknown weathered anthropogenic polymers using this reference library and a commercial Raman database (S&N Labs).

entry no.	specimen	S&N assignment	reference library assignment	match
1	PLAS240	Organic dye and metal oxide pigment	polylactide (polyester)	no
2	PLAS241	polypropylene	polypropylene	yes
3	PLAS244	organic dye	polyvinyl chloride	no
4	PLAS248	HDPE	polyphenylene ether	no
5	PLAS251	polystyrene	polystyrene	yes
6	PLAS252	polypropylene	polypropylene	yes
7	PLAS253	polypropylene and unknown organic additive	pressure sensitive adhesive	Likely overlap - many PSAs contain polypropylene
8	PLAS256	polystyrene	polystyrene	yes

Table S3. Polymers new to open-access spectral repositories.

Polymers new to open-access spectral repositories		
entry	pristine	weathered
1	polyphenylene ether (PPE)	fluorocarbon rubber (FPM)
2	polyvinyl alcohol (PVA)	ultra high molecular weight polystyrene (UHMW)
3	polyethylene cross-linked (PEX)	poly(styrene-butadiene-styrene) (SBS)
4	polybutadiene styrene (PBS)	pressure sensitive adhesive (PSA)
5	polybutylene adipate terephthalate (PBAT)	polybutadiene rubber (BR)
6	polycaprolactone (PCL)	polyoxymethylene (POM)
7	polylactic acid (PLA)	
8	fluorinated ethylene propylene (FEP)	
9	polyether ether ketone (PEEK)	
10	glycol-modified polyethylene terephthalate (PETG)	
11	polyimide (PI)	
12	polyetherimide (PEI)	
13	polyphenylene oxide (PPO)	
14	polytetrafluoroethylene (PTFE)	
15	polyphenylene sulfide (PPS)	
16	polyvinylidene difluoride (PVDF)	
17	perfluoroalkoxyalkane (PFA)	
18	bisphenol-F dihydroxydiphenylmethane (BPF)	
